# Opioid use disorder and role of yoga as an adjunct in management (OUDARYAM): Study protocol for a randomized controlled trial

**DOI:** 10.12688/wellcomeopenres.19392.1

**Published:** 2024-01-03

**Authors:** Hemant Bhargav, Bharath Holla, Jayant Mahadevan, Nishitha Jasti, Mariamma Philip, Priyamvada Sharma, Vedamurthachar A, Urvakhsh Meherwan Mehta, Shivarama Varambally, Ganesan Venkatasubramanian, Prabhat Chand, Gangadhar BN, Kevin P Hill, Nicolas R Bolo, Matcheri Keshavan, Pratima Murthy

**Affiliations:** 1 Department of Integrative Medicine, National Institute of Mental Health and Neurosciences (NIMHANS), Bengaluru, Karnataka, 560029, India; 2 Centre for Addiction Medicine, Department of Psychiatry, National Institute of Mental Health and Neurosciences (NIMHANS), Bengaluru, Karnataka, 560029, India; 3 Department of Biostatistics, National Institute of Mental Health and Neurosciences (NIMHANS), Bengaluru, Karnataka, 560029, India; 4 Department of Clinical Psychopharmacology and Neurotoxicology, National Institute of Mental Health and Neurosciences (NIMHANS), Bengaluru, Karnataka, 560029, India; 5 Department of Psychiatry, National Institute of Mental Health and Neurosciences (NIMHANS), Bengaluru, Karnataka, 560029, India; 6 Division of Addiction Psychiatry, Beth Israel Deaconess Medical Center, Department of Psychiatry, Harvard Medical School, Boston, MA, USA; 7 Neuroimaging in Psychiatry, Department of Psychiatry, Beth Israel Deaconess Medical Centre, Harvard Medical School, Boston, MA, USA; 8 Beth Israel Deaconess Medical Center and Massachusetts Mental Health Center, Harvard Medical School, Boston, MA, USA

**Keywords:** Clinical trials; Yoga; Opioid Use Disorder, functional neuro-imaging, abstinence, mental health.

## Abstract

**Background:**

The proposed research aims to test the effects and mechanisms of a six-month yoga-based intervention as an add-on to standard treatment in opioid use disorder (OUD) by conducting a randomized controlled study with the following primary outcome variables: 1) clinical: abstinence (opioid negative urine test), and reductions in pain and craving, and 2) mechanisms: reward circuit activation in response to opioid visual cue craving paradigm, activation in response to a cognitive control task, and resting state functional connectivity through fMRI, and plasma beta-endorphin levels. Secondary outcome variables are perceived stress, anxiety, sleep quality, cognitive performance, pain threshold, buprenorphine dosage and side effects, withdrawal symptoms, socio-occupational functioning, vedic personality traits, heart rate variability, serum cortisol, and brain GABA levels through magnetic resonance spectroscopy (MRS).

**Methods:**

In this single-blinded, randomized, controlled, parallel-group superiority trial with 1:1 allocation ratio, 164 patients with OUD availing the outpatient/ inpatient clinical services at a tertiary mental healthcare hospital in India will be enrolled after giving informed consent. Consecutive consenting patients will be randomly allotted to one of the two groups – yoga arm (standard treatment + yoga-based intervention), or waitlist group (standard treatment alone). Allocation concealment will be followed, the clinicians, outcome assessors and data analysts will remain blind to subject-group allocation. A validated and standardized yoga program for OUD will be used as an intervention. Participants in the yoga arm will receive 10 supervised in-person sessions of yoga in the initial two weeks followed by tele-yoga sessions thrice a week for the next 22 weeks. The wait-list control group will continue the standard treatment alone for 24 weeks. Assessments will be done at baseline, two weeks, 12 weeks, and 24 weeks. Data from all randomized subjects will be analysed using intent-to-treat analysis and mixed model multivariate analysis.

**Dissemination:**

Findings will be disseminated through peer-reviewed publication, conference presentations, and social media.

**Trial registration number:**

The trial has been registered under Clinical Trials Registry-India with registration number CTRI/2023/03/050737.

## Background

Opioid Use Disorder (OUD) is associated with socio-occupational impairments, financial crisis, and adverse health consequences including high mortality rates (due to overdose and suicide) ^1^. The National Drug Use Survey (2019) reported a prevalence of 2.06% for opioid use in India, with a higher prevalence in urban metropolitan areas ^2, 3^. The COVID-19 pandemic has further inflamed this ongoing ‘hidden epidemic’ in many countries including India ^4^.

Standard treatment for OUD includes pharmacological management with buprenorphine (μ-opioid receptors partial agonist), methadone (μ-opioid receptors agonist) or naltrexone (μ-opioid receptor antagonist) and psycho-social support ^5^. Among them, treatment with buprenorphine or methadone, termed opioid substitution therapy (OST) or medications for opioid use disorders (MOUD) has shown better clinical outcomes. Though generally considered to be safe, the use of OST may be associated with dose-dependent side effects such as respiratory depression, constipation, sleep disturbances, nausea, vomiting, dizziness, tiredness, sweating, sexual dysfunction, and drowsiness ^6, 7^.

A recent systematic review involving 67 studies published in the last 20 years observed that the mean retention rate with OST was 57% at 12 months which further reduced to 38% at three years ^8^. The retention rates reduce even further if the maintenance treatment is with naltrexone or psychosocial support only (in the form of symptomatic treatment with motivational interviewing and relapse prevention treatment) ^9^. Studies have found that adding behavioral therapy did not help in improving retention rates in this population and with retention rate rarely exceeding 50% at 12 months; therefore clinicians have called for innovative and culturally sensitive treatment strategies ^10– 12^. In India, a multi-center study by the All India Institute of Medical Sciences (AIIMS), New Delhi, assessed retention rate with buprenorphine treatment and observed it to be 79% at three months, 70% at six months, and 64% at nine months ^13, 14^. Another large survey in India on 6,600 opioid-dependent participants reported that 67% of OUD patients tried discontinuing treatment on their own in the past year, and only 14% visited de-addiction centers ^1^. Treatment challenges in India include: poor treatment seeking, access barriers, low treatment retention, high relapse rates, lack of personalized, culture, and gender-sensitive interventions, higher cost of medications, and social-stigma associated with treatments ^1, 10^. The major barrier affecting recovery and low treatment seeking was social stigma followed by physical problems ^1^. Similarly, other studies have identified social stigma as the fundamental hindrance in overcoming opioid use crisis ^1, 15, 16^.

The prevalence of chronic pain among adults in India is rising rapidly (19.3% in 2018 as compared to 13% in 2013) ^17^. There is an increasing prescription of opioids for non-cancerous chronic pain, which has also led to increasing non-prescription opioid use. Lack of awareness among prescribers and pharmacists, poor legislation and drug trafficking increase abuse liability ^18, 19^. In patients with chronic pain, greater pain has been shown to predict greater cravings for opioids and subsequent opioid use, and pain coping counseling was shown to suppress this association ^20^. Similarly, mindfulness (an important component of yoga practice) has been shown to help reduce craving in opioid users and this buffering may be associated with improved heart rate variability (HRV) ^21^.

Several recent meta-analyses have reported the beneficial effects of yoga in chronic pain ^22– 25^. Yoga practices enhance sympathovagal balance and reduce stress in psychiatric disorders, and this may also be the biological basis for amelioration of withdrawal symptoms of OUD ^26, 27^. Recent meta-analyses and review articles support the usefulness of yoga as an adjunct in the management of substance use disorders ^28– 31^. Moreover, yoga is free from social stigma and is culturally acceptable and accessible, particularly in the Indian population ^32, 33^. It is also well accepted among female participants ^32^. Interestingly, 34% of the Indian population who suffered from chronic pain, particularly females, opted for yoga and meditation ^17^. Besides this, yoga may enhance pain thresholds ^34^ and bring a state of natural ‘high’ by providing deep relaxation and calmness to the mind. This may be mediated through the enhancement of gamma-aminobutyric acid (GABA; the main inhibitory neuro-transmitter) ^35^, oxytocin ^36^ and endorphin levels ^37, 38^. Yoga has been observed to be as safe as routine physical exercise ^39^.

There are very few studies assessing the effect of yoga in OUD. We could identify only six prospective experimental studies exploring the effects of yoga in OUD patients ^40– 45^. Out of these, three were randomized controlled trials (RCTs) with six months follow-up, two of them demonstrated the utility of yoga in improving quality of life as an add-on to standard treatment. One pilot study (n = 40) showed that add-on Rajayoga meditation led to better improvement in abstinence at six weeks of follow-up than relaxation and standard care or standard care alone ^44^. Another matched controlled study (n = 26) observed add-on yoga to be more effective in reducing perceived stress than standard care alone ^41^. Similarly, a feasibility study found add-on yoga to be feasible and more effective in reducing anxiety and pain, and improving mood than add-on health education after 12 weeks ^45^. None of the previous studies have noted changes in craving, sleep quality, work and social adjustment, withdrawal symptoms, pain threshold, or buprenorphine dosage. Further, there are no studies exploring possible mechanisms of yoga in this population or the option of utilizing tele-yoga for enhancing clinical outcomes in this population.

### Previous work (feasibility and pilot study)

Our team has been working in the area of medical application of holistic yoga-based lifestyle programs for mental and neurological disorders over the last decade. We have validated tools to assess physical and mental constitutions based on traditional Indian medicine ( *prakriti:* physical constitution and *gunas:* mental attributes) for psychiatric patients to design personalized holistic lifestyle programs ^46, 47^. HB has recently developed, validated, and tested the feasibility of a yoga program for OUD (for both the acute withdrawal phase and maintenance phase) ^48^. A functional magnetic resonance imaging (fMRI) paradigm to assess reward circuit activation and craving in response to visual cues related to opioids in OUD patients has been developed (manuscript under preparation). We have been offering tele-yoga services to healthy people for stress management and to patients suffering from mental health disorders for over a year now ^49^. In a pilot feasibility study, we found tele-yoga to be feasible and acceptable in patients with substance use disorders including OUD patients ^50, 51^ and have also observed an increase in plasma endorphin levels immediately after yoga practice in healthy participants ^52^. Tele-medicine offered by the National Institute of Mental Health and Neurosciences (NIMHANS), Bengaluru has been found acceptable and feasible for psychiatric patients ^53^.

In light of the above, a systematic research plan to understand role of long-term yoga intervention as an adjuvant in the management of OUD is proposed.

## Objectives

### Primary objectives

**1. ** To evaluate the effect of a six-month add-on yoga-based intervention on opioid abstinence (opioid-negative urine results and negative self-reported frequency of opioid use since last follow-up), pain and craving in patients with OUD as compared to standard treatment alone.

**2. ** To examine the neurobiological underpinnings of yoga in OUD by estimating plasma beta-endorphins and brain hemodynamics in response to opioid-use-related visual cues, the stop signal task and resting state functional connectivity through fMRI.

### Secondary objectives

**1. ** To evaluate the effect of a six-month add-on yoga-based intervention on perceived stress, pain threshold, sleep quality, cognitive performance (n-back task, Stroop task), work and social adjustment, dosage and side effects of buprenorphine, and yoga-based mental attitudes ( *gunas*).

**2**. To examine the effects of yoga-based intervention on withdrawal symptoms, pain threshold, serum cortisol, brain GABA levels through MRS, heart rate variability and buprenorphine dosage required to score zero on the clinical opiate withdrawal scale (COWS) in patients with OUD during acute withdrawal phase.

### Hypothesis

The yoga-based intervention will serve as a useful adjunct therapy to standard treatment as compared to standard treatment alone in patients with OUD by:

**Primary**-1) improving opioid abstinence, 2) reducing pain and craving, 3) enhancing plasma beta-endorphin levels, and 4) reducing reward system activation in response to visual cue craving paradigm, increasing activation in response to the cognitive control task (stop signal), and increasing coherence in resting state functional connectivity through fMRI.

**Secondary**-1) reducing withdrawal symptoms, disease severity, stress, anxiety, sympathetic arousal as measured by HRV, serum cortisol levels, enhanced brain GABA levels through MRS, cognitive performance (n-back task, Stroop task), reducing dosage and side effects of buprenorphine, and improving sleep quality, pain threshold, and yoga-based mental attitude ( *guna profile-sattva, rajas, tamas*).

**Predictions on mechanisms of action:** We hypothesize that yoga will act through reversing the excitatory/ inhibitory imbalance in the CNS, by increasing GABA and parasympathetic activity, and reducing allostatic load (by reducing cortisol and enhancing beta-endorphins). It will also act by enhancing cognition (enhancing response inhibition with associated activation of prefrontal and anterior cingulate cortices during the stop signal task and improving cognitive performance as measured by n-back task and Stroop task) and by reducing craving as demonstrated by clinical assessments and reducing activation of brain regions that are known to be activated in patients with addiction in response to addiction-related visual cues (prefrontal and orbitofrontal regions, cingulate cortices, inferior frontal regions, limbic system including amygdala and ventral striatum including nucleus accumbens). Yoga may also act by improving functional resting brain connectivity and coherence and enhancing grey matter volume in this population.

## Plan for research

### Trial design

Single-blinded, randomized, controlled, parallel group superiority trial with a 1:1 allocation ratio.

### Sample size estimation

For evaluating the primary objectives, the optimal sample size was estimated using the principles and methods for the assessment of primary parameters (abstinence, pain, craving, plasma beta-endorphins and fMRI changes). Based on approximation and estimation from the previous data on the clinical effects of yoga interventions in OUD/other patients with substance use disorder supporting a medium effect size ^37, 40, 43, 44^, it was estimated that, for an allocation ratio of one, a sample size of at least 82 OUD patients in each arm (accounting for a potential drop-out rate of 36% at six months ^40^ with 60 completing the entire study (for the pre- versus post- comparisons of patients) will be required to detect a two-tailed significant difference of α = 0.01 with an estimated 80% power.

The subset sample size of 50 for imaging studies (in each group – pre as well as post-brain scans) will be sufficient to perform random effect analysis for fMRI BOLD changes with multiple comparison correction using random field theory-based family-wise error correction to control for multiple comparison ( http://www.fil.ion.ucl.ac.uk/spm/).

### Study setting

**Figure 1.**
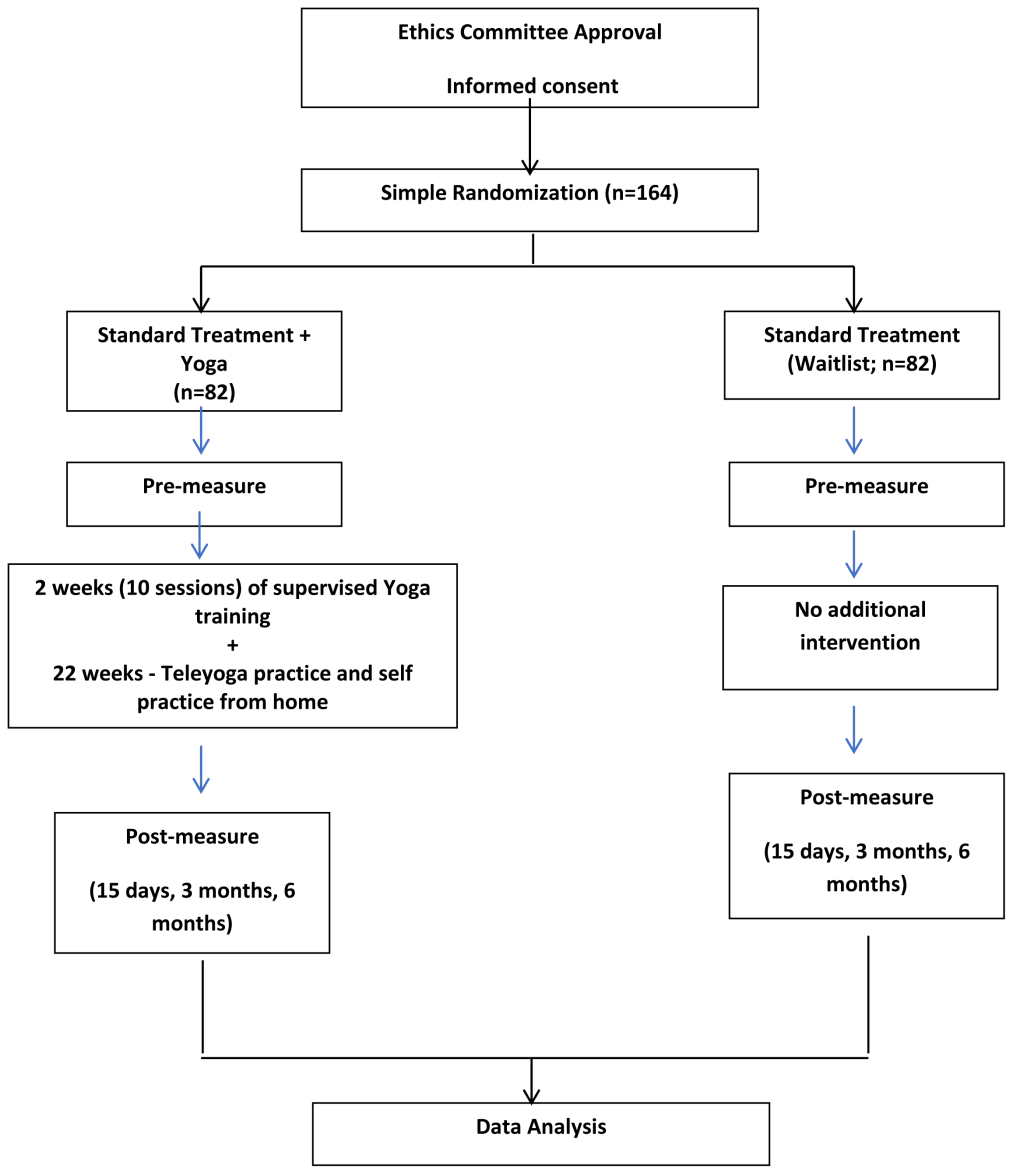
Study design outline.

Patients with OUD availing the outpatient/ inpatient clinical services under the Centre for Addiction Medicine, Department of Psychiatry, National Institute of Mental Health and Neurosciences (NIMHANS), Bengaluru, India will be approached. The course of the trial, its benefits, possible demerits, voluntary participation with a choice to quit the study at any time will be explained in simple comprehensible language. Patients satisfying the inclusion criteria will be requested to provide written informed consent. Consecutive consenting patients will be randomly assigned to one of the two groups – Standard treatment + Yoga-based intervention (YAT), or waitlist group-standard treatment (TAU) alone by the biostatistician. Allocation sequence will be generated by biostatistician using computer-generated random numbers and allocation concealment will be done using opaque sealed envelopes. A validated and standardized yoga program for OUD will be used as an intervention. Although double blinding will not be possible in the study, the clinicians treating the subjects, the outcome assessors, and data analysts will remain blind to the subject-group allocation status throughout the period of the study. All participants will be continuing their routine standard care (OST/MOUD and psycho-social support) till the study concludes. Figure 1 provides the study design outline. Buprenorphine/naloxone dosage will be recorded at the beginning of yoga intervention; dosage will be adjusted based on the clinical presentation of the patient by the treating psychiatrist.

### Selection of participants

***Inclusion criteria***

a) Diagnosis of OUD (DSM 5 criteria)
b) Score >3 on Clinical Global Impression – Severity (CGI-S)
c) Age range: 18–50 years
d) All gender
e) Written informed consent
f) Access to smartphone and internet

***Exclusion criteria***

a) Prescription of OST other than buprenorphine/naloxone
b) Severe withdrawal symptoms (COWS>25)
c) Other diseases causing intractable pain
d) Prescription opioids for pain related to other medical conditions
e) Co-morbid alcohol/benzodiazepine/cannabis/multiple substance dependence in last six months except for nicotine
f) Impaired intellectual functioning (HMSE>24)
g) MRI contraindications such as claustrophobia, metal implants or paramagnetic objects in the body
h) Neurosurgery/loss of consciousness due to head injury in the past/diseases affecting CNS
i) Severe psychiatric or medical illness preventing practice of the yoga program
j) Pregnancy or post-partum
k) Risk of harm to self or others
l) Current/ recent practice of yoga/meditation (>4hr/month in last three months)

### Clinical assessment

The demographic and clinical information will be collected using structured scales and proforma. Diagnosis of OUD will be made as per Diagnostic and Statistical Manual of Mental Disorders (DSM 5) criteria and participants will be screened using Diagnostic Interview for Anxiety, Mood, and OCD and Related Neuropsychiatric Disorders (DIAMOND) ^54^. The clinical diagnosis would also be confirmed independently by a qualified psychiatrist. Pain and Craving will be assessed by Brief Pain Inventory (BPI) and Opioid Craving Scale (OCS), respectively. Withdrawal symptoms will be assessed by Clinical Opiate Withdrawal Scale (COWS). The Time-Line Follow Back (TLFB) method will be employed at the three- and six-month intervals to quantify self-reported frequency of opioid use and maximum weeks of abstinence. The TLFB is a reliable and valid tool in assessing self-reported drug use and leverages a calendar and significant events to aid participants in recalling their substance use patterns, thereby offering a comprehensive view of consumption trends over time ^55^. Perceived stress will be assessed using the perceived stress scale (PSS). Work and social life will be rated on the Work and Social Adjustment Scale (WSAS) and Quality of Sleep on Pittsburg Quality of Sleep Index (PSQI). Adverse effects of opioid substitution therapy (buprenorphine/naloxone) will be assessed using the Udvalg for Kliniske Undersogelser (UKU) Side Effects Rating Scale.

In addition, traditional diagnoses related to yoga therapy will be determined at baseline using the Vedic Personality Inventory and Ayu-Soft Prakriti Diagnostic Tool. Table 1 provides the schedule of assessment.

**Table 1.** Schedule of assessments.

Assessments	Day 0	15 Days/10 Sessions	Three Months	Six Months
**Clinical Assessment**				
DSM-5 Clinical Interview Diagnosis	✓			
DIAMOND	✓			
Urine Toxicology for Opiods (monthly)	✓	✓	✓	✓
Self-reported Opioid Use Frequency (TLFB)	✓	✓	✓	✓
Maximum Consecutive Abstinence Weeks (TLFB)	✓		✓	✓
**Pain and Craving Assessment**				
Brief Pain Inventory	✓	✓	✓	✓
Opioid Craving Scale	✓	✓	✓	✓
**Physiological Measures**				
Plasma Beta-Endorphin Levels	✓		✓	✓
Functional Magnetic Resonance Imaging (visual cue craving task, stop signal task, resting state functional connectivity and MRS)	✓		✓	✓
Heart Rate Variability	✓	✓		
Pressure Algometer (Pain Threshold)	✓	✓	✓	✓
Serum Cortisol	✓		✓	✓
**Mental Health and Quality of Life Measures**				
Pittsburgh Quality of Sleep Index	✓		✓	✓
Perceived Stress Scale	✓		✓	✓
Clinical Global Impression (Severity & Improvement)	✓	✓	✓	✓
Cognitive Performance (n-back task, Stroop task)	✓		✓	✓
Work and Social Adjustment Scale (WSAS)	✓		✓	✓
**Medication and Withdrawal Measures**				
Udvalg for Kliniske Undersogelser (UKU) Side Effects Rating Scale + Visual Analog Scale	✓	✓	✓	✓
Clinical Opiate Withdrawal Scale (COWS)	✓	✓	✓	✓
Buprenorphine Dosage	✓	✓	✓	✓
Fagerstrom Test for Nicotine Dependence	✓	✓	✓	✓
**Yoga Related Measures**				
Vedic Personality Inventory	✓			✓
Yoga Performance Assessment (By Trained Therapist)	✓	✓	✓	✓

**Key: "✓" - Assessment will be conducted**. Abbreviations: TLFB = Time-Line Follow Back method; MRS = Magnetic Resonance Spectroscopy

### Data storage, handling, and statistical analysis

A data monitoring committee (DMC) will be created, which will be chaired by the biostatistician. The DMC will be independent from the sponsor and any competing interests. Data handling, coding and password-protected data storage will be supervised by a biostatistician. The biostatistician will also supervise data anonymization and data analysis. The data will be suitably anonymized so that no identification features are revealed. Data anonymization will be done by the biostatistician creating a mirror image of a database and implementing alteration strategies, such as character shuffling or encryption.

**Primary outcome measures:** Key measures include opioid abstinence confirmed by negative urine screening and self-report, levels of pain measured by BPI, the intensity of craving tracked by OCS, beta-endorphin levels, and fMRI-BOLD responses to tasks (stop signal, visual cue craving) and resting state functional connectivity through fMRI. 

**Secondary outcome measures:** These include percentage of participants maintaining abstinence, the longest duration of continuous abstinence, withdrawal symptoms (COWS), severity (CGI), pain threshold (algometer), cognitive performance (n-back task and Stroop task), stress levels (PSS), quality of sleep (PSQI), functioning (WSAS), HRV, cortisol levels, brain GABA levels through MRS, buprenorphine dosage during withdrawal and maintenance phases, side effects (UKU), and gunas (VPI).

### Data analysis

The R programming language will be used to analyze data (R Core Team 2022; 56). Details of dropped-out/withdrawn patients will be recorded. Missing data, drop-outs, non-compliance, and adverse events will be managed in collaboration with the biostatistician and supervisor. The data of all randomized participants will be analyzed using intent-to-treat analysis and mixed model multivariate analysis. Imaging analysis details are provided below. OST/MOUD dosage will be used as a covariate in the analysis. Interim analysis will be done when 50% of the target sample size completes the trial. Information regarding solicited and spontaneously reported adverse events and other unintended effects of trial interventions or trial conduct will be documented and reported to the DMC as well as the ethics committee of the institution. The DMC will have access to interim analysis results and will take the decision to terminate the trial in case of unreasonable morbidities or mortalities among the recruited participants during the trial. Information obtained from the interim analysis may be used for enhancing trial efficiency by updating design and/or sample size adjustments, this will be done as per the suggestions of DMC and after due approval from the funding agency. Trial conduct will be audited once in a year by the DMC, the process will be independent from investigators and the sponsor.

### Intervention

**Yoga-add on therapy (YAT: Standard treatment + yoga)**: Based on our previous clinical experience the duration of intervention was decided to be 24 weeks ^48– 51^. Yoga will be offered as an add-on to standard treatment. Participants will be trained in a 40-minute validated yoga module (30 minutes practice+10 minutes relaxation) ^48^ during the withdrawal phase and a 60-minute yoga module (45 minutes practice+15 minutes relaxation) during maintenance phase by a trained yoga-instructor (MSc Yoga Science) at the Department of Integrative Medicine, NIMHANS Bengaluru. Table 2 and Table 3 provide the details of the yoga practices to be used during withdrawal and maintenance phase, respectively. Initial supervised training will be conducted for two weeks (five sessions/week). Participants will be asked to join supervised tele-yoga practice from home for the next 22 weeks (at least three days/week). A monthly direct supervised booster session along with yoga-practice booklet, logbook and practice video will be provided. A mobile phone software application will be used to share yoga videos with the participants. Yoga compliance and fidelity to the yoga module (when it has to be done by oneself using video) will also be assessed. Patients will also be monitored by phone calls. Yoga performance will be monitored through the yoga performance assessment scale (YPA). 

**Table 2.** Yoga module for OUD in the acute phase of withdrawal - 30 Minutes of practice + 10 minutes of relaxation.

Step	Yoga Practices	Position	No of Cycles*	Duration (Total 40 Minutes)
	Starting prayer (prayer for health and well-being)	Sitting	1	1 min
**Physical Postures and Practices (Asanas)**				
1	Instant relaxation technique, rapid tightening of body parts from toes to head	Supine	2	4 min
2	Straight leg raising, using alternate legs	Supine	5	3 min
3	Pavanamuktasana (wind releasing pose), using alternate legs	Supine	5	2 min
4	Makarasana (crocodile pose), using alternate legs	Prone	5	3 min
**Breathing Practices (Pranayama)**				
1	Deep abdominal breathing with prolonged exhalation; inhalation: exhalation = 1:2 and movement with awareness on different points or joints of the body from head to toe and back	Supine	5	2 min
2	Vibhagiya, sectional breathing, Chin Mudra—breath in: hold: breath out = 4:16:8	Sitting	5	2 min
3	Vibhagiya, sectional breathing, Chinmaya Mudra—breath in: hold: breath out = 4:16:8	Sitting	5	2 min
4	Vibhagiya, sectional breathing, third, Adi Mudra—breath in: hold: breath out = 4:16:8	Sitting	5	2 min
5	Bhastrika slow without hand movements, each cycle interspersed with rest for 30 seconds (bellows breathing)	Sitting	2 sets of 10 strokes each	3 min
6	Nadi Shuddhi (alternate nostril breathing,)	Sitting	9	3 min
7	Bhramari (humming breath) preferably in Shanmukhi Mudra	Sitting/ supine	9	3 min
**Meditative and Relaxation Practices (Dhyana)**				
1	Guided yogic relaxation in Shavasansa (corpse pose), with positive affirmation—slow part-by-part relaxation of body parts with imagery from toes to head, with chanting of “OM” or humming “MMM” for 9 cycles and gradual expansion of non-judgmental awareness and positive affirmation: “I am not the body, I am not the mind, I am the observer - free, relaxed, and satisfied” at the end.	Supine	1	9 mins
2	End, prayer for universal peace	Sitting	1	1 min

**Table 3.** Yoga module for OUD in the maintenance phase - 45 Minutes of practice + 15 minutes of relaxation.

Step	Yoga of Practices	Position	No of Cycles*	Duration
	Starting prayer (prayer for health and well-being)	Sitting	1	1 min
**Physical Postures and Practices (Asanas)**				
1	Instant relaxation technique, rapid tightening of body parts from toes to head	Supine	2	4 min
2	Straight leg raising, using alternate legs followed by both legs	Supine	5 each leg	2 min
3	Pavanamuktasana Kriya (wind releasing pose), using alternate legs	Supine	5 rounds clockwise, 5 rounds anticlockwise	5 min
4	Makarasana (crocodile pose), using alternate legs followed by both legs	Prone	5	2 min
5	Bhujangasana breathing (Cobra pose)	Prone	5	2 min
6	Naukasana (boat pose), using alternate hands and feet followed by both	Prone	5	2 min
7	Patangasana (butterfly pose) using both legs	Sitting	100 strokes; 1 cycle	2 min
8	Vyaghra Shwasa (tiger stretch pose)	Sitting on knees	5	2 min
9	Surya Namaskara (sun salutations), 10 steps each round with 4 cycles slow and 4 cycles fast	Standing	8 rounds	8 min
**Breathing Practices (Pranayama)**				
1	Deep abdominal breathing with prolonged exhalation; inhalation : exhalation = 1:2 and movement of awareness on different points/joints of the body from head to toe and back	Supine	5	1 min
2	Vibhagiya, sectional breathing: Chin Mudra (breath in : hold : breath out = 4:16:8)	Sitting	5	2 min
3	Vibhagiya, sectional breathing: Chinmaya Mudra (breath in : hold : breath out = 4:16:8)	Sitting	5	2 min
4	Vibhagiya, sectional breathing: Adi Mudra (breath in : hold : breath out = 4:16:8)	Sitting	5	2 min
5	Bhastrika – medium 1 cycle, fast 1 cycle and slow 1 cycle interspersed with rest for 30 seconds (bellows breathing)	Sitting	3 sets of 20 strokes each, with gap of 30 seconds	3 min
6	Nadi shuddhi (alternate nostril breathing)	Sitting	9	3 min
7	Bhramari (humming breath) in Shanmukhi Mudra	Sitting/ supine	9	2 min
**Meditative and Relaxation Practices (Dhyana)**				
8	Guided yogic relaxation in Shavasansa (corpse pose), with positive affirmation—slow part-by-part relaxation of body parts with imagery from toes to head, with chanting of “OM” or humming “MMM” for 9 cycles and gradual expansion of non-judgmental awareness and positive affirmation: “I am not the body, I am not the mind, I am the observer - free, relaxed, and satisfied” at the end.	Supine	1	14 mins
9	End, prayer for universal peace	Sitting	1	1 min
10	Discussions from yoga philosophy perspective: happy analysis *(ananda* * mimamsa)* and inquiry into the nature of the “self” *(aatma parikasha)*	Once a week for 10 minutes		

**Standard treatment-as-usual (TAU waitlist) group:** They will continue to take prescribed medications (OST/MOUD) and psychosocial support as prescribed by the psychiatrist. They will be requested to appear for the planned assessments and will be offered yoga after the six-month study period.

The allocated intervention may be discontinued or modified in case of any subject reporting worsening of symptoms. This data will be documented.

***Description of some major assessments***

**1)**  **Urine screening for opiods:** Urine screening for opioids is considered as a gold standard for monitoring abstinence. Test depending on the Immunoassay method will be used. It will be done using the opioid strip test which is a rapid, easy and reliable test that is routinely used in clinical settings in India.
**2)**  **Brief Pain Inventory ^57^:** It is a reliable and valid tool to assess pain. The scale measures pain intensity, pain severity and pain interference. It has good internal consistency (Cronbach’s alpha = 0.87). The scale is rated by the patient, has 16 items, and provides details regarding pain in the last 24 hours.
**3)**  **Opioid craving scale ^58^:** It is a three-item test that is a reliable and valid tool to assess craving in opioid use disorders ^59^ and has been used in many studies before. It has good internal consistency (omega = 0.81). Each item is rated by the patient on a visual analog scale from 0–10.
**4)**  **Plasma beta-endorphin estimation:** A blood sample will be collected around 9 am after overnight fasting. 10ml of blood will be drawn and collected into chilled EDTA (1ml/ml of blood) tubes containing aprotinin (500 KIU/ml). The sample will be centrifuged at 1600 × g for 15 minutes at 2 – 8°C. The plasma sample will be immediately frozen and kept at -80°C till analysis. Plasma beta-endorphin levels will be measured using MILLIPLEX MAP multiplex assay kit (Millipore Life sciences, MA), and analyzed by LUMINEX xMAP technology. (CV=6.25%).
**5)**  **Serum Cortisol estimation:** Venous blood (around 5 ml) will be collected from patients in anticoagulant-free tubes around 9 am and serum will be separated after 30 minutes by centrifugation (2900 rpm for 15 minutes). Coded serum sample will be stored at -80°C. The serum cortisol levels will be measured using Enzyme-linked Immuno-Sorbent Assay (ELISA) by commercial reagents according to the manufacturer's instructions.
**6)**  **Magnetic Resonance Imaging (MRI):**
**Data Acquisition:** Scans will be executed on a Philips 3T Ingenia CX MRI equipped with a 32-channel proton RF head coil. The cumulative duration for all scanning protocols is estimated to be approximately 50 minutes. Functional imaging will employ a T2*-weighted echo-planar sequence during both resting-state (seven min) and two task paradigms: the Stop-Signal Task (SST, 12 min) and opioid-cue reactivity (12 min).
  The whole brain will be covered with axial slices of 3 mm thickness, acquired in descending order (37 slices, 25% gap). Parameters: TR=2000 ms, TE=30 ms, flip angle=78°, matrix=64×64, and FOV=192×192 mm ^2^. The first four volumes per run will be discarded. Structural sequences will include T1- (6 min), and T2-weighted (three min). Parameters: T1 – TR/TE (ms) = 6.5/2.9; flip angle = 9°, acquisition matrix = 256×256, and FOV = 256 ×256×192 mm ^2^; T2- TR/TE (ms) = 3000/80; flip angle = 90°, acquisition matrix = 240×165, and FOV = 149.5 ×240×176 mm ^2^.
**Resting State fMRI**: Participants will be instructed to keep their eyes open, stare at a cross on the screen, and avoid purposeful mental activity during a seven-minute resting-state session. This session is intended to capture intrinsic neural activity patterns in the absence of directed cognitive tasks, allowing for the assessment of various resting-state networks and functional connectivity across brain regions.
**Task fMRI:** Stimuli will be displayed via a plastic mirror mounted on the head coil using E-prime software. **Stop-Signal Task**: Using an event-related design, this task comprises two runs, each with 180 trials over six minutes. Participants will respond to arrows pointing left or right, displayed against a mid-grey background. They are directed to indicate the arrow's direction as promptly and accurately as possible using a two-button panel aligned with their dominant hand. Notably, 16.67% of these trials are ‘Stop’ trials, wherein an upward arrow unexpectedly appears for 300ms post the directional arrow. The task aims to examine the ability to inhibit responses, facilitated by the higher prevalence of ‘Go’ trials which establish a dominant ‘Go’ response tendency. A dynamic tracking algorithm will adjust the Stop Signal Delay to balance successful and unsuccessful inhibitions at the stop trials, aiming for a 50-50 ratio. This cognitive task will be followed by **Opioid-cue Reactivity task:** This 12-minute task comprises 12 blocks (four neutral (N), four drug-related (D), and four blank (B) blocks) displayed in a pseudo-random sequence. Each block lasts for 30 seconds and features five images shown for six seconds each. Drug-related images are assorted into four sub-categories: tablets, cough syrup, scenes of drug chasing, and intravenous drug use. The task aims to capture neural responses associated with opioid cue-reactivity and quantify alterations in cue reactivity subsequent to intervention.
**Data Preprocessing and Analysis:** The acquired scans will be analyzed using AFNI (Analysis of Functional Neuro Images) software. Preprocessing of T1w and fMRI data will be performed using @SSwarper and afni_proc.py respectively. The @SSwarper command will nonlinearly warp each subject's T1w anatomical dataset to the India Brain Template (IBT) standard space ^60^. Within afni_proc.py, functional data will undergo realignment, slice time correction, co-registration to the T1w image using the LPC+ZZ cost function, spatial warping to IBT space using estimated nonlinear warps and finally spatial smoothing of 6 mm (FWHM) by an isotropic Gaussian Kernel.
  For resting-state scans, independent component analysis will be conducted using the GIFT toolbox to segregate neural networks. In the case of the Stop-Signal Task (SST), an event-related design will be implemented to contrast Stop-Success and Stop-Failure conditions, serving as a measure of impulsivity. For the opioid-cue reactivity paradigm, our primary interest lies in the drug-neutral cue contrast.
  AFNI’s 3dREMLfit will be utilized to perform generalized least squares (GLSQ) modeling with a restricted maximum likelihood (REML) approach, accounting for temporal correlation. The resulting effect estimates from 3dREMLfit for the contrast conditions from the subject level will subsequently be transferred to the group level via the AFNI program 3dLME. The group will be modelled as a between-subject factor with two levels (TAU and YAT), while time (0, 3, and 6 months) will be treated as a within-subject variable and participants as random factors. A cluster-level family-wise error (FWE) rate corrected significance threshold will be computed to correct for multiple comparisons. This calculation will be undertaken using 3dClustSim, factoring in the smoothness of the residuals, which will be estimated with the -acf option inside the gray matter mask using the 3dFWHMx tool. The thresholds derived will subsequently be applied to the statistical maps produced by 3dLME, aiding in the identification of significant clusters.

## MRS Using MEGAPRESS for Brain GABA Quantification

To supplement our fMRI investigations, we will employ proton Magnetic Resonance Spectroscopy (MRS) utilizing the MEGAPRESS (MEscher-GArwood Point-Resolved Echo Spectroscopy Sequence) difference-editing technique in a small subset of the recruited population. This sequence is tailored for the isolation and quantification of GABA concentration.

The MRS acquisition will target the left thalamus with voxel dimensions of 2×2×3 cm. The focus on the left thalamus is informed by extant literature that identifies this region as having enhanced parasympathetic innervations and reduced GABA levels in participants experiencing anxiety, a fundamental symptom prevalent in opioid withdrawal and relapse scenarios.

Manual voxel shimming will be employed to achieve global water-line widths less than 15 Hz. The MEGAPRESS difference-editing sequence will be utilized for this protocol. Key parameters include a repetition time (TR) of 2 seconds, an echo time (TE) of 68 milliseconds, and a spectral bandwidth of 2 kHz. The readout duration will be 512 milliseconds with the number of excitations (NEX) set at 384, leading to a total acquisition time of 13 minutes. The specifically tuned GABA editing pulse will be applied in an interleaved fashion on alternate transients.

**Ethical statement:** The proposed study has been approved by the Institutional Ethics Committee (No.NIMHANS/HECAIM/6th/Meeting 2021–22 dated 13th September, 2022) and registered with CTRI. Written Informed consent will be taken from all participants by the investigator, and study records will be kept confidential. Subject identifying information will be replaced with codes. Additional consent will be taken for collection and use of participant data and biological specimens in ancillary studies. Ethics and dissemination norms will be followed as per SPIRIT 2013 checklist ( https://www.spirit-statement.org/spirit-statement/). Major amendments to the protocol (changes to eligibility criteria, outcomes, analyses) will be communicated to investigators, institutional ethics committee, trial registries and the funding agency.

**Compensation for loss of time/wages:** A gift-card with a value of Rs. 1000 will be given to all the participants at assessments 0, two weeks, three months, and six months to compensate for lost work-hours. Travel of the participants for the supervised yoga sessions or for assessments will be covered from the grant. A yoga mat will be given to participants for practicing yoga at home.

**Insurance:** All participants in the study will be insured for health problems. This is necessary as per Institutional Ethics Committee norms.

**Implications:** This prospective randomized trial will attempt to replicate and confirm the utility of Yoga in the clinical management of OUD, which has worldwide relevance. The study may also help establish a neurobiological basis for the effects of Yoga in substance use disorders.

**Plans for data management, sharing and dissemination of research findings:** As the clinical and imaging data acquired through this study is likely to be unique in terms of its comprehensive coverage of various neurobiological parameters that can potentially facilitate the understanding of mechanistic basis of yoga, in conformation with the policy of Wellcome Trust–DBT India Alliance on data sharing, necessary efforts will be made to maximize public benefit. As early as possible, as per the ethical norms suggested by the institutional ethics committee, the researchers will make the anonymized data widely available to the research community so that they can be verified, built upon and used to advance knowledge. The policy for sharing data will be in tune with good research practice guidelines. To facilitate further research studies on neurobiology of yoga, the study findings will be communicated to the user communities. Ethical usage of data sharing with good research practice form consent will be obtained by the sharing researchers. NIMHANS also has a Digital Academy, a Tele-medicine Centre and a Department of Mental Health Education that deal with the dissemination of information to social media. These platforms will be used to disseminate results of this study.

**Roles and Responsibilities:** HB, PM, SSV, BNG, BH and MK designed the study. JM and PC will help in patient recruitment. KH, JM, and PC will supervise clinical assessments. KH will provide clinical inputs while designing fMRI tasks. HB and NJ will help in training the yoga therapist who will deliver the intervention. PC will help in developing a digital platform for delivering the yoga program. MP will supervise randomization, data management and statistical analysis. PS will supervise technical aspects related to blood parameters. BH, UM and GS will help fMRI protocol optimization and fMRI data analysis. NRB will supervise fMRI and MRS data extraction and analysis. MK will supervise clinical and fMRI related training outside the host-institution.

## Dissemination policy

Results of the trial will be communicated to research committee and policy makers by publishing them in peer reviewed scientific journals. Subsequently, media platforms will be used to communicate study findings to general population. Authorship eligibility will be decided as per the contribution made by the author towards the trial. No professional writers will be used to write the manuscripts. Protocol will be published in a journal that provides free public access.

## Data Availability

No data are associated with this article. OSF: SPIRIT checklist and flow chart for ‘Opioid use disorder and role of yoga as an adjunct in management (OUDARYAM): Study protocol for a randomized controlled trial. https://osf.io/829pa ^61^.
