# Supplementary material for: Opioid use disorder and role of yoga as an adjunct in management (OUDARYAM): Study protocol for a randomized controlled trial
Source: Wellcome Open Res. Author manuscript; Available in PMC 2025 Jun 16. (PMC11249518; doi:10.12688/wellcomeopenres.19392.1)
Supplement: Supplementary Material [file EMS205869-supplement-Supplementary_Material.pdf]

Open Peer Review

Current Peer Review Status: ✔ ✔

Version 1

Reviewer Report 15 July 2024

https://doi.org/10.21956/wellcomeopenres.21480.r81281

© **2024 Deepeshwar S.** This is an open access peer review report distributed under the terms of the Creative Commons Attribution License, which permits unrestricted use, distribution, and reproduction in any medium, provided the original work is properly cited.

✔ **Singh Deepeshwar**

Babasaheb Bhimrao Ambedkar University, Lucknow, Uttar Pradesh, India

Dear Author,
The manuscript "Opioid use disorder and role of yoga as an adjunct in management (OUDARYAM): Study protocol for a randomized controlled trial" presents a timely and significant investigation into the potential benefits of integrating yoga into the treatment of opioid use disorder. This study protocol is well-designed, demonstrating a thorough understanding of the complexities involved in managing opioid dependence and the innovative potential of complementary therapies. By exploring the adjunctive role of yoga, this research has the potential to offer valuable insights into holistic and integrative approaches to addiction treatment, potentially improving patient outcomes and quality of life. The rigorous methodological approach outlined promises to contribute meaningful data to the field, advancing both scientific knowledge and clinical practice.

However, a few specific comments on the protocol are as follows:

1. Highlight the rationale for using two yoga modules with different practice times.

2. Please add details of urine screening for opioids. Which time of the day will this test be done?

3. Please add rationale of using Stop-signal task in the study.

Is the rationale for, and objectives of, the study clearly described?

Yes

Is the study design appropriate for the research question?

Yes

Are sufficient details of the methods provided to allow replication by others?

Yes

Are the datasets clearly presented in a useable and accessible format?
Yes

Competing Interests: No competing interests were disclosed.

Reviewer Expertise: Yoga and Cognitive Neuroscience and have engaged in yoga research for the for the last 15 years.

I confirm that I have read this submission and believe that I have an appropriate level of expertise to confirm that it is of an acceptable scientific standard.

Reviewer Report 04 June 2024

https://doi.org/10.21956/wellcomeopenres.21480.r80298

© 2024 Krishnan A. This is an open access peer review report distributed under the terms of the Creative Commons Attribution License, which permits unrestricted use, distribution, and reproduction in any medium, provided the original work is properly cited.

Ameya Krishnan
Yale University, New Haven, Connecticut, USA

The article is scientifically sound and compelling. I have no corrections / revisions to add at this time. I think this research, once implemented, could set an exciting precedent for further study and understanding of the neurobiology of yoga and yoga-derived practices, in a manner that is more comprehensive than in the extant literature on this topic. Appreciate the authors' thorough protocol development and explication of the same.

Is the rationale for, and objectives of, the study clearly described?
Yes

Is the study design appropriate for the research question?
Yes

Are sufficient details of the methods provided to allow replication by others?
Yes

Are the datasets clearly presented in a useable and accessible format?
Not applicable

Competing Interests: No competing interests were disclosed.

I confirm that I have read this submission and believe that I have an appropriate level of expertise to confirm that it is of an acceptable scientific standard.
